# A Case of Acute Cholecystitis Successfully Treated With Endoscopic Ultrasound-Guided Gallbladder Drainage in a Patient With Osteogenesis Imperfecta

**DOI:** 10.7759/cureus.48424

**Published:** 2023-11-07

**Authors:** Koichiro Mandai, Koji Uno

**Affiliations:** 1 Department of Gastroenterology, Kyoto Second Red Cross Hospital, Kyoto, JPN

**Keywords:** drainage, gallbladder, endoscopic ultrasound, acute cholecystitis, osteogenesis imperfecta

## Abstract

A 74-year-old man with severe osteogenesis imperfecta (OI) was admitted to our hospital because of repetitive cholecystitis due to a stone in the gallbladder neck. Because he had severe OI-related chest wall deformity and a high risk of complications from bronchial intubation, general anesthesia, and surgery, we performed endoscopic ultrasound-guided gallbladder drainage (EUS-GBD). The postprocedural clinical course was uneventful, and he was discharged in satisfactory condition. EUS-GBD is a treatment option for acute cholecystitis in surgically high-risk patients with OI. However, special attention should be paid to the influence of sedation on the respiratory and cardiovascular systems during the procedure.

## Introduction

Osteogenesis imperfecta (OI) is a genetic bone fragility disorder characterized by low bone mass, skeletal deformity, and variable short stature. Despite significant variability in clinical features and severity within OI [1], individuals with severe OI often exhibit chest wall deformities and spinal issues, presenting a heightened risk of complications during bronchial intubation, general anesthesia, and surgery.

Recently, the efficacy of endoscopic ultrasound-guided gallbladder drainage (EUS-GBD) for acute cholecystitis has been reported in patients with difficult surgery [2]. EUS-GBD is a transmural drainage method in which a stent is placed from the gallbladder to the stomach or duodenum under EUS and fluoroscopic guidance.

## Case presentation

**Figure 1:**
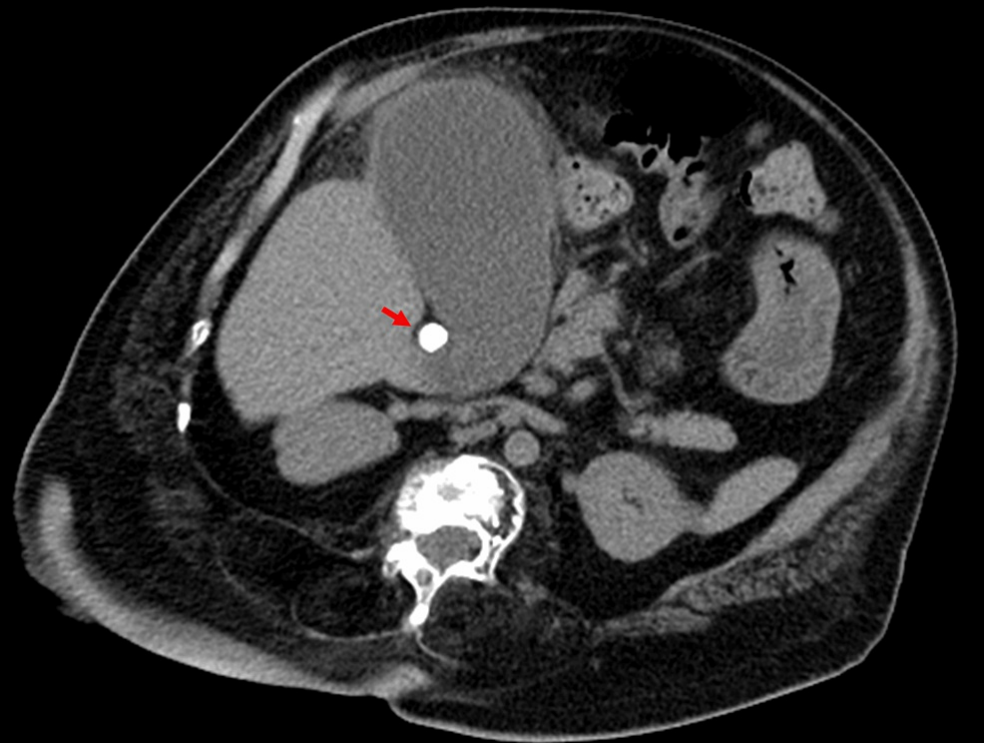
Computed tomography image on admission The gallbladder was swollen and its wall was thickened. An impacted stone can be seen at the gallbladder neck (arrow).

A 74-year-old man (height 120 cm) with severe OI presented at the emergency room of our hospital because of fever and right upper abdominal pain. He had been hospitalized for acute cholecystitis and was discharged two weeks earlier. He had undergone home oxygen therapy for chronic pulmonary dysfunction caused by a chest wall deformity. He could not walk independently and had been in a wheelchair due to the deformation and shortening of the lower limbs. He was febrile (37.8°C) and had right upper abdominal tenderness. Laboratory data showed a normal white blood cell count of 7100/mm3 (normal range, /mm3) but an elevation of C-reactive protein (12.97 mg/dL; normal range, 0.00-0.40 mg/dL). Abdominal computed tomography (CT) showed swelling and wall thickening of the gallbladder and an impacted stone located at the gallbladder neck (Figure 1).

**Figure 2:**
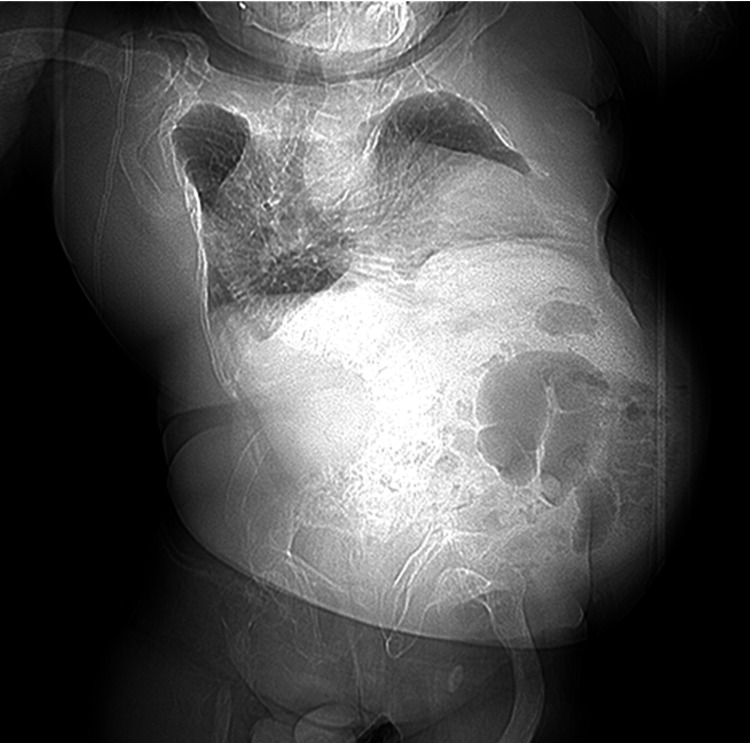
Radiography image The chest wall and spine of the patient were severely deformed.

We made a diagnosis of acute cholecystitis due to a gallbladder stone. Before this time, he had experienced acute cholecystitis five times. However, he had low pulmonary function caused by the deformity of the chest wall and spine due to OI (Figure 2).

He also had a high risk of complications from bronchial intubation, general anesthesia, and surgery. Moreover, percutaneous transhepatic gallbladder drainage was impossible because of the spine deformity. We believed that acute cholecystitis could reoccur owing to the impacted stone, even if the patient recovered with antibiotic therapy. Therefore, we decided to perform EUS-GBD after he provided informed consent.

**Figure 3:**
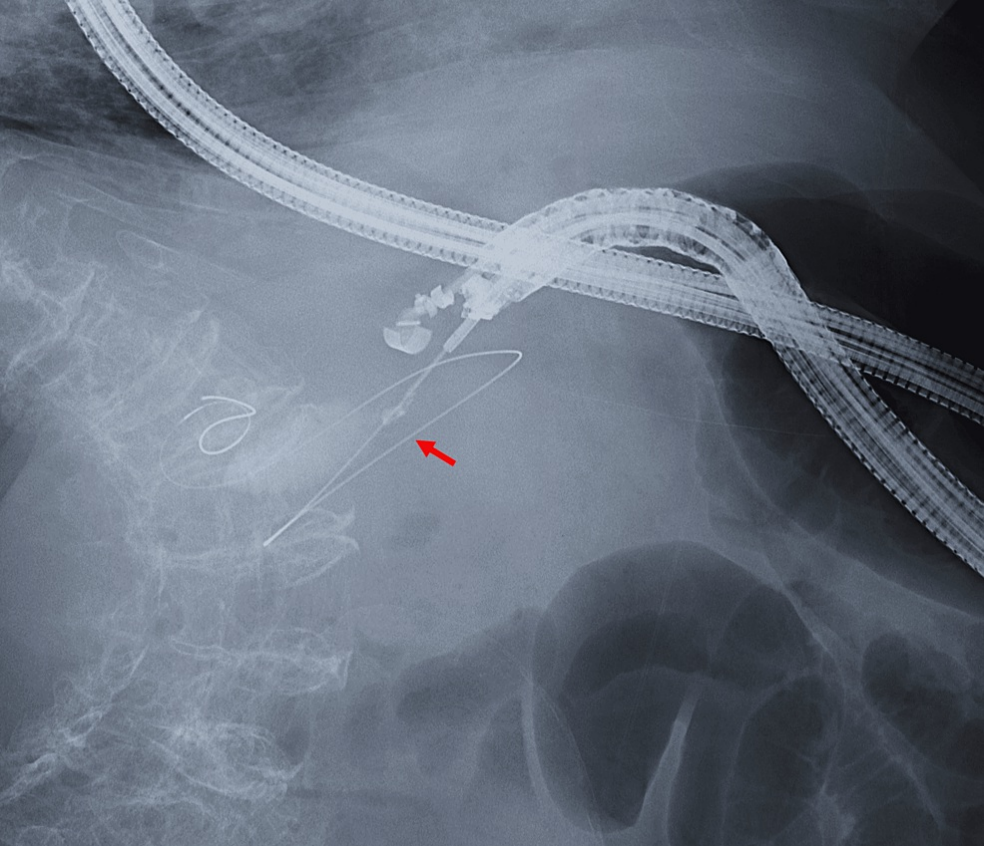
Fluoroscopy image during EUS-GBD A 0.025-inch guidewire (arrow) was introduced through the needle and coiled into the gallbladder. EUS-GBD: endoscopic ultrasound-guided gallbladder drainage

On the third hospital day, we performed EUS-GBD. The procedure was performed in the slight left lateral position because the patient could not maintain the prone position or spine position due to the chest wall deformity. To avoid respiratory depression, we did not use midazolam for sedation but used pethidine and dexmedetomidine. First, pethidine was slowly injected intravenously at a dose of 35 mg, and an infusion of 4 μg/kg/h of dexmedetomidine was administered for 10 min. Thereafter, a continuous infusion of 0.4 μg/kg/h of dexmedetomidine was started and continued. The procedure was performed using a convex-type echoendoscope (GF-UCT260; Olympus Medical Systems, Tokyo, Japan). After the echoendoscope was introduced into the duodenum, the gallbladder was visualized. A 19-gauge needle (EZShot3 Plus; Olympus Medical Systems) was used to puncture the gallbladder, and a 0.025-inch guidewire (VisiGlide; Olympus Medical Systems) was introduced through the needle and coiled into the gallbladder under fluoroscopic guidance (Figure 3).

**Figure 4:**
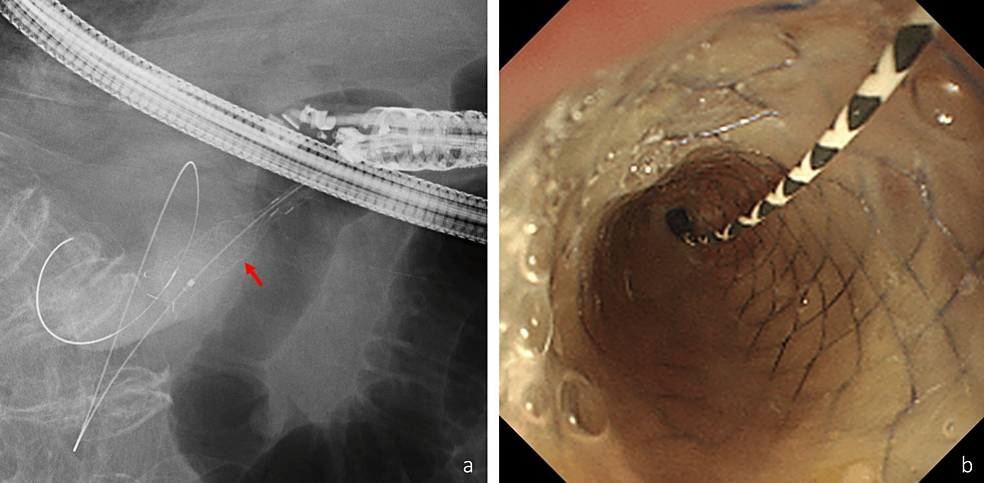
Fluoroscopy image (a) and endoscopic image (b) A fully covered metal stent (arrow) was placed from the gallbladder to the duodenum.

The fistula was dilated using a 4-mm balloon catheter (REN; Kaneka Medix, Osaka, Japan), and a fully covered metal stent with a diameter of 10 mm and length of 6 cm (BONA stent; Standard SciTech Inc., Seoul, Korea) was placed from the gallbladder into the duodenum (Figure 4a, 4b).

Finally, a 7-Fr double pigtail biliary plastic stent with a length of 10 cm (Mediglobe GmbH, Rosenheim, Germany) was placed from the gallbladder to the duodenum through the metal stent to prevent stent migration and food impaction. Abnormal respiratory and circulatory suppression related to the sedation was not observed during the procedure.

**Figure 5:**
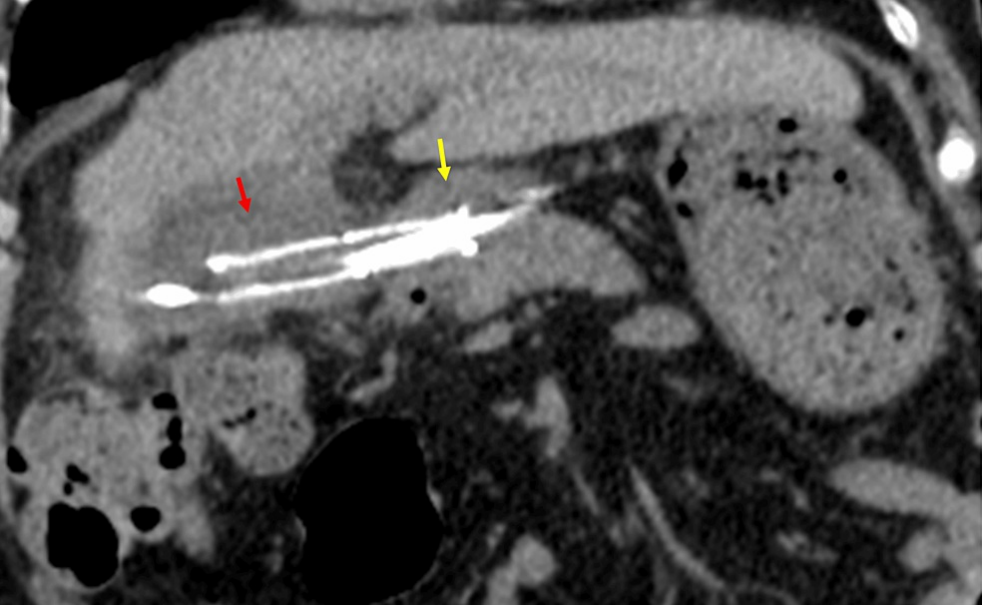
Computed tomography of the coronal view on post-EUS-GBD day seven The stent was located from the gallbladder (red arrow) to the duodenum (yellow arrow). EUS-GBD: endoscopic ultrasound-guided gallbladder drainage

The clinical course after EUS-GBD was uneventful, and CT on post-EUS-GBD day seven did not show stent migration or dislocation (Figure 5).

The patient was discharged without stent removal on post-EUS-GBD day ten and has had no adverse events three months after discharge.

## Discussion

OI is a clinically heterogeneous, heritable connective tissue disorder linked directly to type I collagen. Its common clinical features encompass low bone mass and diminished bone material strength, rendering individuals prone to bone fragility, fractures, bone deformities, and growth deficiencies. Occurring in approximately 1/15,000-20,000 births [3], OI is classified into five types: I) non-deforming OI with blue sclera; II) perinatally lethal OI; III) progressively deforming OI; IV) common variable OI with normal sclera; and V) OI with calcification of the interosseous membranes [4].

Our patient was diagnosed with type III OI, evident through short stature and severe chest wall and spine deformities, leading to compromised pulmonary function. These structural issues posed a high risk of complications during bronchial intubation, general anesthesia, and surgery.

In high-risk surgical cases of acute cholecystitis, percutaneous transhepatic gallbladder drainage is typically considered the first alternative therapy [5]. However, in our patient, a spine deformity made this approach unfeasible. Endoscopic transpapillary gallbladder drainage was deemed challenging due to a stone impacted in the gallbladder neck, leading us to opt for EUS-GBD. The procedure proved effective, and the patient was discharged without complications.

While midazolam is a commonly used sedative for therapeutic endoscopy, in this case, we employed dexmedetomidine due to its respiratory safety profile [6,7]. Dexmedetomidine demonstrated efficacy in sedation without observable abnormal respiratory or circulatory depression during the procedure.

## Conclusions

We present a case of acute cholecystitis successfully treated with EUS-GBD in a patient with severe OI. We consider that EUS-GBD under sedation with dexmedetomidine is a treatment option for acute cholecystitis in surgically high-risk patients with OI, although special attention should be paid to the influence of sedation on the respiratory and cardiovascular systems.
